# Retinal Vessel Segmentation by Deep Residual Learning with Wide Activation

**DOI:** 10.1155/2020/8822407

**Published:** 2020-10-10

**Authors:** Yuliang Ma, Xue Li, Xiaopeng Duan, Yun Peng, Yingchun Zhang

**Affiliations:** ^1^Institute of Intelligent Control and Robotics, Hangzhou Dianzi University, Hangzhou 310018, Zhejiang, China; ^2^Key Laboratory of Brain Machine Collaborative Intelligence of Zhejiang Province, Hangzhou 310018, Zhejiang, China; ^3^Department of Biomedical Engineering, University of Houston, Houston 77204, TX, USA

## Abstract

**Purpose:**

Retinal blood vessel image segmentation is an important step in ophthalmological analysis. However, it is difficult to segment small vessels accurately because of low contrast and complex feature information of blood vessels. The objective of this study is to develop an improved retinal blood vessel segmentation structure (WA-Net) to overcome these challenges.

**Methods:**

This paper mainly focuses on the width of deep learning. The channels of the ResNet block were broadened to propagate more low-level features, and the identity mapping pathway was slimmed to maintain parameter complexity. A residual atrous spatial pyramid module was used to capture the retinal vessels at various scales. We applied weight normalization to eliminate the impacts of the mini-batch and improve segmentation accuracy. The experiments were performed on the DRIVE and STARE datasets. To show the generalizability of WA-Net, we performed cross-training between datasets.

**Results:**

The global accuracy and specificity within datasets were 95.66% and 96.45% and 98.13% and 98.71%, respectively. The accuracy and area under the curve of the interdataset diverged only by 1%∼2% compared with the performance of the corresponding intradataset.

**Conclusion:**

All the results show that WA-Net extracts more detailed blood vessels and shows superior performance on retinal blood vessel segmentation tasks.

## 1. Introduction

Image segmentation in retinal blood vessels has an important medical application value [1]. Analyzing the changes of the retinal vascular structure is a key part in diagnosing retinal diseases such as diabetic retinopathy (DR) [2], hypertension, and arteriosclerosis. However, the retinal blood vessels show complex network structures and low contrast, causing difficulties in manual segmentation.

Many methods have been proposed in recent years by researchers in the field. Broadly, these methods can be divided into two groups: unsupervised and supervised approaches [3]. In unsupervised approaches, main algorithms include matched filtering [4], morphological processing [5], vascular tracking [6], and model-based [7]. However, an extreme limitation of these unsupervised methods is that they heavily rely on handcrafted features for vessel representation and segmentation. Besides, the parameters need to be elaborately designed. Therefore, unsupervised methods are inferior to supervised approaches in terms of segmentation accuracy.

Supervised learning, different from unsupervised methods, requires manual annotations to build the optimally predictive model. There are two processors which are needed: one is an extractor, and the other one is a classifier. The features of the retinal vessel can be extracted by Gabor filter [8], Gaussian filter [9], and so on. In traditional machine learning, K-nearest neighbor (K-NN), SVM, AdaBoost, etc. are often used to train the classifier [10–12]. In the study conducted by Zhu et al. [13], filters, morphological operators, and linear detection operators were used to extract retinal vascular morphological features as prior information for the classifier. Based on a fully connected conditional random field (CRF) model, Orlando et al. [14] used a structured output support vector machine to learn model parameters and perform retinal vessel segmentation. For these traditional supervised methods, the final results predicted are greatly influenced by the features used for classification. However, they are often defined empirically, which prevents the generalization of the unknown geometric transformation of blood vessels.

Given the aforementioned limitations, it remains a challenging task to effectively and automatically segment retinal blood vessels that warrants further studies using novel techniques. Deep learning, with powerful data representation capabilities, has been widely used in retinal image segmentation tasks. Fu et al. [15] formulated the vessel segmentation to a holistically-nested edge detection (HED) problem and utilized the fully convolutional network (FCN) to generate the vessel probability map. Mo and Zhang [16] developed a deep supervised FCN by leveraging multilevel hierarchical features. Liskowski and Krawiec [17] regarded blood vessel segmentation as a binary classification task, using convolutional neural network (CNN) to perform pixel-by-pixel classification, and combined structured prediction methods to classify multiple pixels at the same time. The results obtained are better than most traditional algorithms. However, the network has tens of millions of parameters. Recently, it is more challenging to develop a simpler but effective model to accurately segment small vessels. Jin et al. [18] proposed deformable U-Net (DUNet), which integrated deformable convolution blocks, to detect the tiny vessels. It has fewer parameters but longer calculation time. Gu et al. [19] developed the context encoder network (CE-Net) to preserve spatial information for medical image segmentation. However, its segmentation accuracy on the vessel segmentation task is unsatisfactory. Azad et al. [20] took full advantages of U-Net [21], dense convolutions [22], and bidirectional ConvLSTM (BConvLSTM) [23] to segment vessels. They achieved a higher accuracy, but excessive use of dense block (DB) consumes memory greatly. Budak et al. [24], in their recent work, showed that the weak and thin vessels are better segmented by the cascading CNN of lesser depth as long as feature utilization is improved. For this reason, and motivated by the fact that wide activation, which focuses on the width of deep learning, propagates more low-level features with the same parameter complexity [25], in this study, we develop a retinal vessel segmentation method by deep residual learning based on wide activation.

The remaining of this paper is organized as follows: Section 2 presents the proposed method, Section 3 provides implementation details, Section 4 analyzes the experimental results, and discussion and conclusion are drawn in Section 5 and Section 6, respectively.

## 2. Proposed Method

**Figure 1:**
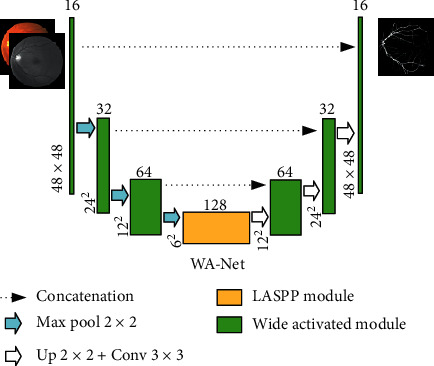
Retinal vessel image segmentation model. The numbers above each block stand for the input filters. At the lower left edge are the dimensions of each block. In the legend, Concatenation means the merger of channels, Max pool denotes max pooling, Up indicates upsampling, and Conv represents convolution. The number following them is the kernel size.

An overview of the proposed segmentation model is shown in Figure 1. The original retinal image is preprocessed and input to WA-Net, and then the segmented image is output after network mapping. This framework has two main modules, denoted as wide activated module and LASPP module, which will be introduced in the following parts.

### 2.1. Wide Activated Module

In the traditional convolutional neural network, the output of the *l*^th^ layer can be calculated by the following equation:

(1) $$\begin{array}{c} x_{l}=H_{l}\left(x_{l-1}\right), \end{array}$$

where  *x*_*l*_  denotes the output of the *l*^th^ layer, *x*_*l*−1_ denotes the output of the former (*l*-1)^th^ layer, and *H* denotes a convolution often activated with ReLU, where ReLU is defined as *f*(*x*_*l*−1_)=max(0, *x*_*l*−1_).

In order to make full use of features, He et al. [26] proposed residual mapping to build a deeper network. The definition of residual mapping is as follows:

(2) $$\begin{array}{c} F\left(x_{l}\right)=Y\left(x_{l}\right)-x_{l}, \end{array}$$

where *x*_*l*_ is the input, *Y*(*x*_*l*_) is the output mapping, and *F*(*x*_*l*_) is the residual mapping.

**Figure 2:**
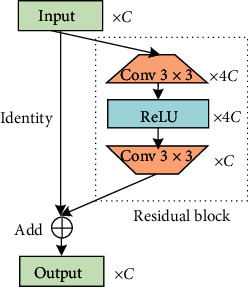
Schematic of WDSR-A. Identity represents the identity mapping layer, Add denotes the element-wise summation over channels. In the dashed box, Conv stands for convolution, ReLU indicates the activation layer, *C* is the abbreviation of the channel, and the two conical shapes are the convolution layers that expand and slim the channel, respectively. The principle of wide activation is expanding features before the activation layer without increasing computation. A further explanation is shown in Figure 3.

Wide activated module (WDSR-A) [25] is a kind of residual module which is based on wide activation as shown in Figure 2. It mainly consists of four layers: two convolution layers, an activation layer (ReLU) and an identity layer. In the practical experiment, a 1 × 1 convolution is used in the identity layer when channels do not match.

#### 2.1.1. Original Residual Block

**Figure 3:**
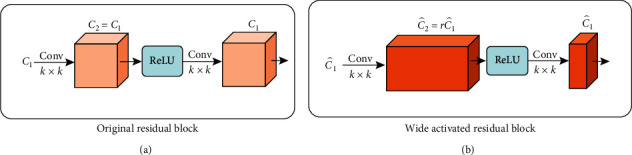
Principle of wide activation [25]. *C*_*i*_ and ${\hat{C}}_{i},\;i=1,2$, stand for channels, *r* is the expansion factor, and *k* × *k* below Conv is the kernel size. In deep learning, the width refers to the number of channels. Wide activation focuses on the width (the *C* parameter) to improve feature utilization.

For the original residual block (Figure 3(a)), suppose the width of the identity mapping pathway is *c*_1_ and the width before activation in the residual block is *c*_2_; then, *c*_2_=*c*_1_, so the parameters in each original residual block are 2 × *c*_1_^2^ × *k*^2^, where *k* is the kernel size.

#### 2.1.2. Wide Activated Residual Block

Figure 3(b) shows the residual block with wide activation. It is calculated by the following equation:

(3) $$\begin{array}{c} {\hat{c}}_{2}=r\times {\hat{c}}_{1}, \end{array}$$

where *r* is the expansion factor before activation and ${\hat{c}}_{1}\;$ and ${\hat{c}}_{2}$ are the width of the identity mapping pathway in WDSR-A and the width before ReLU, respectively.

When the patch size of the input is fixed, the computational complexity is a fixed proportion of the parameters. In order to make WDSR-A maintain the same complexity as the original residual block, the following equation is established:

(4) $$\begin{array}{c} c_{1}^{2}={\hat{c}}_{1}\times {\hat{c}}_{2}=r\times {\hat{c}}_{1}^{2}. \end{array}$$

According to the above equation, the width of the identity mapping pathway in WDSR-A needs to be slimmed by factor $\sqrt{r}$, and the width before activation should be expanded by $\sqrt{r}$. This paper takes *r* = 4.

### 2.2. LASPP Module

A major challenge in vessel segmentation is how to capture tiny blood vessel features. This problem was solved by atrous convolution [27], which allows to enlarge the receptive field without increasing parameters.

**Figure 4:**
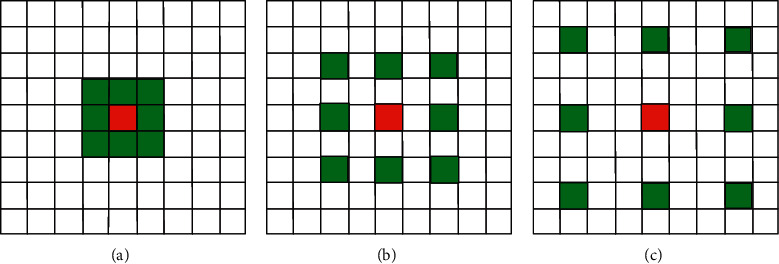
Schematic of atrous convolutions. (a) *d* = 1. (b) *d* = 2. (c) *d* = 3.

The principle of the atrous convolution is to insert 0 pixels between each pixel of the traditional convolutional kernel, i. e., to increase the dilation rate *d* of the network. For each pixel *i* of the output *y*, the process of the atrous convolution is expressed as

(5) $$\begin{array}{c} y\left(i\right)=\sum_{k}x\left(i+d\times k\right)w\left(k\right), \end{array}$$

where *w*(*k*) denotes a filter, *k* is the filter size, *x* denotes the input, and *d* is the dilation rate. As shown in Figure 4, the size of the convolution kernel is 3 × 3, and Figures 4(a)–4(c) correspond to atrous convolutions of *d* = 1, *d* = 2, and *d* = 3, respectively.

Different dilation rates can be used to change the receptive field. For the atrous convolutional layer with the dilation rate of *d* and kernel size of *k*, the receptive field size is calculated by the following equation::

(6) $$\begin{array}{c} R_{f}=\left(k-1\right)\times \left(d-1\right)+k. \end{array}$$

For example, if a convolutional kernel size is 3 × 3 with dilation rate *d* = 3, the corresponding receptive field size is 7. The superposition of multiple convolutional layers allows for a greater receptive field.

**Figure 5:**
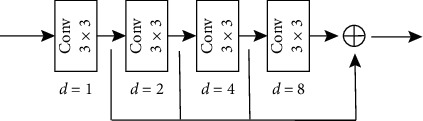
Schematic of the LASPP module.

Atrous spatial pyramid pooling (ASPP) [28] is a model based on the atrous convolution. It adopts several parallel convolutional layers with different expansion rates to improve the segmentation performance. Inspired by this, at the bottom of the network, we designed a module like ASPP (LASPP) to preserve multiscale features of blood vessels, as shown in Figure 5. The dilation rates used in the four convolutional layers are *d*=2^*i*^,  *i*=0,1,2,3, with kernel size 3 × 3 and activated by ReLU. Finally, the features extracted with different dilation rates are added to generate the fusion result and send to the decoding structure.

### 2.3. Weight Normalization

In training deep neural networks, batch normalization (BN) is frequently used after each convolution to solve the internal covariate shift problem [29]. However, BN has the drawback of data dependence on the mini-batch [25]. In this way, we prefer to use weight normalization (WN) to speed up the convergence speed of the network and improve the accuracy of training and testing [30].

Weight normalization, in simple terms, is the reparameterization of weight vectors in the CNN. Assume the output **y** has the following form:

(7) $$\begin{array}{c} y=w\cdot x+b, \end{array}$$

where **w** is a *k*-dimensional weight vector, *b* denotes a scalar bias term, and **x** is a *k*-dimensional vector of the input features. WN reparameterizes the weight vectors with new parameters by the following equation::

(8) $$\begin{array}{c} w=\frac{g}{\left|\left|N\right|\right|}N, \end{array}$$

where **N** is a *k*-dimensional vector, *g* denotes a scalar, and ||**N**|| is the Euclidean norm of **N**. With this formalization, we will have ||**w**|| = *g*, which is independent of parameter **N** [30].

### 2.4. Network Structure: WA-Net

**Figure 6:**
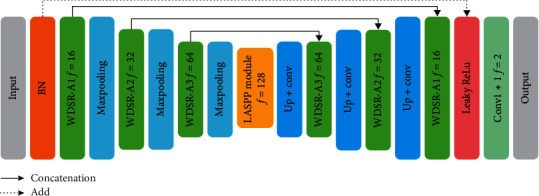
WA-Net structure.

Figure 6 illustrates the wide activation network (WA-Net). The architecture consists of two parts: encoder and decoder. For the encoder part, the image patches are input to the network for batch normalization (only one time). Then, each WDSR-A module is followed by a 2 × 2 max pooling layer. In the decoding phase, Up + conv means that the upsampling was followed by a 3 × 3 convolution without ReLU. The dashed line in Figure 6 represents a global shortcut with a convolution of 1 × 1 and leaky ReLU. Behind WDSR-A1 in the decoding section is another leaky ReLU. We add the outputs of the two leaky ReLUs and put them into a 1 × 1 × 2 convolution layer with softmax to do classification.

The motivation of leaky ReLU is to avoid zero gradients. It is defined as follows:

(9) $$\begin{array}{c} f\left(x_{i}\right)=\left\{\begin{array}{cc} x_{i}, & \text{if }x_{i}>0,\\ a_{i}x_{i}, & \text{if }x_{i}\leq 0, \end{array}\right.\; \end{array}$$

where *x*_*i*_ is the input on the *i*^th^ channel and *a*_*i*_ is a coefficient controlling the slope of the negative part. It degenerates into ReLU when *a*_*i*_=0. This paper sets *a*_*i*_=0.3 for leaky ReLU. The specific parameter settings of each module are shown in Table 1.

### 2.5. Loss Function

In retinal images, the distribution of blood vessels and non-blood vessels is unbalanced. In order to solve this problem, a combined loss function is adopted. It can be defined by the following equation:

(10) $$\begin{array}{c} L\left(\bar{y},y\right)=L_{\text{CE}}\left(\bar{y},y\right)+L_{\text{DICE}}\left(\bar{y},y\right), \end{array}$$

where $L_{\text{CE}}\left(\bar{y},y\right)$ denotes cross-entropy, which is defined as follows:

(11) $$\begin{array}{c} L_{\text{CE}}\left(\bar{y},y\right)=-\sum_{i}\left(y_{i}\log\bar{y_{i}}+\left(1-y_{i}\right)\log\left(1-\bar{y_{i}}\right)\right). \end{array}$$

And $L_{\text{DICE}}\left(\bar{y},y\right)$ denotes a loss function based on the dice coefficient [31]. It is defined by the following equation:

(12) $$\begin{array}{c} L_{\text{DICE}}\left(\bar{y},y\right)=1-\frac{2\sum_{i}y_{i}\bar{y_{i}}+k}{\sum_{i}y_{i}+\sum_{i}\bar{y_{i}}+k}, \end{array}$$

where *i* is the number of pixels, *y*_*i*_  is the ground truth, and$\;\bar{y_{i}}\;$ is the predicted result, respectively. *k* is a smooth value set to 1.0 to correct the function.

## 3. Implementation

The network model was implemented under PyCharm simulation platform using Python 3.6 with Tensorflow1.13. All experiments were conducted in a 64 bit Windows 10 laptop with Intel Core i7-8750H CPU @ 2.20 GHz 2.21 GHz, 16 GB RAM, NVIDIA GeForce GTX 1050Ti GPU.

### 3.1. Data and Data Preprocessing

The fundus images used in the experiment are from two public datasets: DRIVE (Digital Retinal Images for Vessel Extraction) [10] and STARE (Structured Analysis of the Retina) [32]. There are 40 images in the DRIVE dataset with a resolution of 565 × 584, while the STARE dataset consists of 20 images with a pixel size of 605 × 700. Experts' manual labels in both datasets are available as the ground truth.

**Figure 7:**
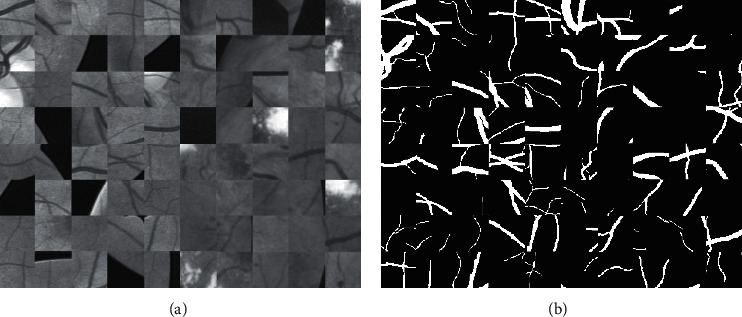
Patches sampled for model training: (a) the preprocessed patches; (b) the corresponding ground truth.

In the deep convolutional neural network, appropriate preprocessing allows better segmentation of retinal blood vessels. In this study, all images were preprocessed as follows: firstly, the RGB images were converted into grayscale and standardized. Then, contrast-limited adaptive histogram equalization (CLAHE), which is a method of image enhancement, was used to improve the brightness and contrast of the image [33]. To further improve image quality, we introduced gamma correction, where *γ* = 1.2. The preprocessed images were divided into local overlapping patches by the sliding window with 48 × 48 and the stride size of 5. The patches sampled randomly and preprocessed are shown in Figure 7.

### 3.2. Training Details

Adam [34] is a common optimizer that speeds up network convergence. The proposed network selected Adam as the optimizer with initial learning rate 0.001, and *β*_1_=0.9, *β*_2_=0.999, and *ε*=10^−8^. The batch size and epochs were set to 32 and 100, respectively. A kernel weight initialization method named as He normal, which is proposed by He et al. [35], was used to initialize the kernel weights of the WDSR-A modules.

As for the division of datasets, the DRIVE dataset has been divided into the training set (20 images) and testing set (the rest 20 images) fixedly. Considering that the STARE dataset does not explicitly provide the training and testing sets, this paper selects the first 10 images as training images and the rest 10 as test images according to Wang et al. [36]. In this study, we used the patch-based strategy to reduce overfitting. During the training process, the DRIVE and STARE datasets were divided into 160,000 and 200,000 patches, respectively, of which 90% patches were selected for training, and the other 10% were used as validation.

### 3.3. Performance Evaluation

In order to quantitatively evaluate the blood vessel segmentation effect, 5 evaluation metrics were used, including accuracy (Acc), sensitivity (Sens), specificity (Spec), *F*1 score (*F*1), and area under the curve (AUC). The first 4 evaluation indicators are defined as

(13) $$\begin{array}{c} \text{Acc}=\;\frac{T_{P}+T_{N}}{T_{P}+F_{P}+T_{N}+F_{N}},\\ \text{Sens}=\frac{T_{P}}{T_{P}+F_{N}},\\ \text{Spec}=\frac{T_{N}}{T_{N}+F_{P}},\\ \text{Prec}=\frac{T_{P}}{T_{P}+F_{P}},\\ F1=2\times \text{Prec}\times \frac{\text{Sens}}{\text{Prec}+\text{Sens}}, \end{array}$$

where *T*_*P* _ is true positive, indicating the correctly segmented vascular pixels, *T*_*N*_  is true negative, standing for the correctly segmented nonvascular pixels, *F*_*P*_  is false positive, denoting incorrectly segmented vascular pixels, and *F*_*N*_  is false negative, denoting incorrectly segmented nonvascular pixels.

The receiver operating characteristic (ROC) curve is also an important curve to measure the effect of retinal blood vessel segmentation. The closer the area under the curve (AUC) is to 1, the more accurate a model is.

## 4. Experimental Results

### 4.1. Vessel Segmentation Results

**Figure 8:**
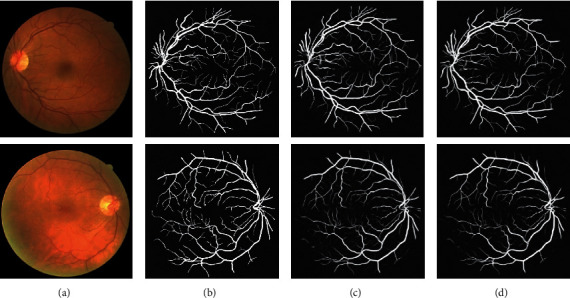
Comparison of the segmentation results on the DRIVE dataset: (a) original image; (b) ground truth; (c) U-Net; (d) proposed WA-Net.

**Figure 9:**
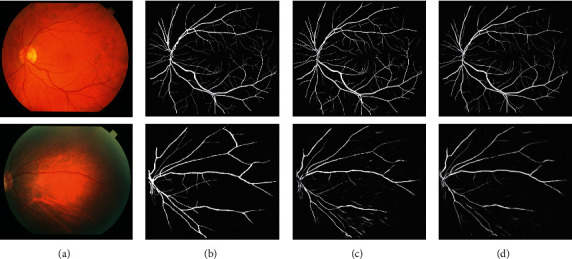
Comparison of the segmentation results on the STARE dataset: (a) original image; (b) ground truth; (c) U-Net; (d) proposed WA-Net.

The proposed WA-Net was compared to custom U-Net on DRIVE and STARE datasets. Figures 8 and 9 are the segmentation results. In these figures, the first row shows healthy retinal images, while the second shows unhealthy retinal images. As can be seen from Figures 8 and 9, the segmentation results were consistent with those of experts and even better on some small vascular branches. In addition, the noise and erroneous segmentation were reduced. Furthermore, WA-Net obtained desirable segmentation results in those weak vessels. On the DRIVE dataset, WA-Net had fewer segmentation errors and enhanced for some small vessel segmentation. On the STARE dataset, it is worth noting that the segmentation errors were significantly reduced. On the other hand, for the healthy group, WA-Net had less vascular noise and more continuity. For the unhealthy group, U-Net was more prone to mis-segmentation. These results proved that the proposed network had a better segmentation than U-Net.

### 4.2. Comparison with Other Methods

In order to further verify the performance of WA-Net in retinal blood vessel segmentation, this paper compared the evaluation metrics with existing methods on DRIVE and STARE datasets, respectively. Tables 2 and 3 present the global performance of the methods used in different literature studies on the test set.

Generally speaking, the performance of unsupervised methods is not as effective as supervised approaches. As can be seen from Table 2, on the DRIVE dataset, *F*1, Sens, Spec, Acc, and AUC of WA-Net were 0.8222, 0.7875, 0.9813, 0.9566, and 0.9794, respectively, which were superior to most algorithms. *F*1 obtained the highest value, and Sens was only slightly lower than CE-Net. For Acc and AUC, DCCMED-Net and WA-Net achieved the best results, which further verify the effectiveness of increasing feature utilization. Acc of WA-Net was slightly lower than that of DCCMED-Net due to the latter network cascaded three encoder-decoder modules, while WA-Net used one. However, DCCMED-Net in Sens was dramatically lower than WA-Net 6.11%. This phenomenon also appeared on WA-Net of Table 3. A possible explanation might be the extreme imbalance of blood vessels and nonvessels. In retinal images, the pixels of the blood vessels only occupy a small proportion of the entire image. When they achieve a high global accuracy, a small number of mis-segmented pixels will have a greater impact on blood vessel pixels but less impact on background pixels. Therefore, the sensitivity is relatively low.

As shown in Table 3, Spec and Acc of WA-Net achieved the highest on the STARE dataset. Dense U-Net outperformed WA-Net 1.74% in Sens, but Spec, Acc, and AUC of WA-Net were 1.49%, 1.07%, and 1.21% higher than Dense U-Net. To sum up, by comparing all the listed results, WA-Net obtained competitive performances on DRIVE and STARE datasets.

### 4.3. Segmentation Results between Datasets

**Figure 10:**
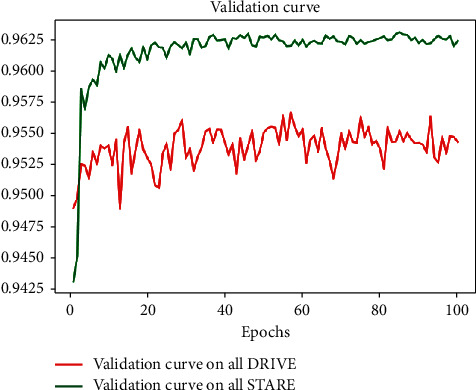
Validation curve on two datasets.

In our work, we proved the generalization ability of the proposed network. Considering the DRIVE and STARE datasets were obtained using two devices with obviously different physical resolutions, this paper verified a more demanding scenario where one dataset was used for training and another for testing. Verification was performed on both DRIVE and STARE datasets, with the verification curve shown in Figure 10.

The tested AUC and Acc indicators of cross-training are summarized in Table 4. As listed in Table 4, when tested on the STARE dataset, we obtained the highest AUC, while Acc was lower than Yan et al. [42] slightly. However, Acc and AUC tested on the DRIVE dataset were undesirable. One possible explanation might be that the DRIVE dataset comprises many thin blood vessels, while the training set (STARE) mainly contains thick blood vessels [18]. On the other hand, we compared the performance within datasets (Tables 2 and 3) and found that the performance of Acc and AUC between datasets diverged only 1%∼2% compared with the intradatasets. All these results demonstrated generalizability of WA-Net.

### 4.4. The Influence of Network Structures

**Figure 11:**
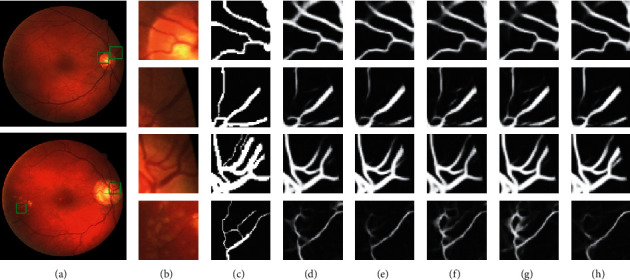
Magnified patches of different network structures on DRIVE: (a) original image; (b) magnified RGB patch; (c) ground truth; (d) Network_1; (e) Network_2; (f) Network_3; (g) Network_4; (h) WA-Net.

**Figure 12:**
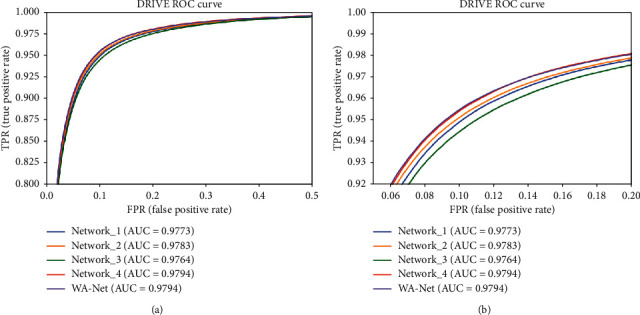
ROC curves of different structures on DRIVE: (a) ROC curves; (b) zoomed ROC curves on the top left of (a).

This section investigated the effects of using the wide activation structure and atrous convolution. We adjusted WA-Net and presented the performance indicators on the DRIVE dataset. The wide activated module in WA-Net was replaced with the preactivated residual module [46]: BN-ReLU-Conv ⟶ BN-ReLU-Conv, and LASPP module removed WN, named as Network_1. Based on Network_1, according to the traditional CNN, channels were set to 32-64-128-256-128-64-32, named as Network_2. Based on WA-Net, reduce the number of layers in the original LASPP module to 3 as Network_3, and then increase layers to 5, where *d* = 16 of the 5th layer, named as Network_4. The details of these networks are presented in Figure 11. Furthermore, ROC curves of different structures are shown in Figure 12. The closer the ROC curve to the top-left border is in the ROC coordinates, the more accurate a model is.

Figure 11 shows that the thick blood vessel branches could be segmented by all adjusted networks, but WA-Net greatly reduced mis-segmentation. For complicated vessels, it is difficult for the network to separate small blood vessels when the thick and thin vessels are close to each other. At the junction of thick blood vessels, more information can be extracted by WA-Net, while Network_3 did not detect it at all. On multiconnected small blood vessels, it is difficult for the segmentation algorithms to proceed precisely due to the local lesion. In summary, WA-Net is able to distinguish different vessels and present a better performance than the other networks.

## 5. Discussion

The difficulty in segmenting retinal blood vessels accurately from RGB images mainly arises from their low pixel intensity, which makes them (especially some tiny vessels) similar to the background and results in difficult segmentation. To address this issue, we proposed WA-Net based on wide activation. With the help of the widened channels, more vessel information was transmitted to the subsequent atrous convolutional layers. Then, convolutional layers superimposed with different dilation ratios are used to capture contextual weak blood vessel information of different sizes more effectively. Due to the difficulty in recovering some low-level information, skip layer connection is utilized to directly fuse low-level information and high-level information in the network structure. Comparison of the results obtained with several methods and the proposed algorithm on the DRIVE and STARE datasets shows that the segmentation accuracy by WA-Net has led to a higher level as shown in Tables 2 and 3. In terms of the algorithm itself, it is expected that it will be robust and accurate. Therefore, cross-training between datasets is performed in Table 4, which verified the generalization performance of the model. Utilizing the characteristic that the number of feature channels is doubled at each downsampling step, the slimmed identity mapping path reduced the parameters of WA-Net.

Despite the improvement achieved in this study, there are still several limitations. Considering that the deep learning segmentation method could produce the most accurate results when there are sufficient labeled data, while fewer samples are there in retinal images, a more effective data augmentation can be achieved using the generative adversarial network (GAN). In addition, although the parameters of WA-Net are reduced, there is still a long computational time due to the introduction of weight normalization. Further investigation on how to effectively reduce the calculation time is required.

The segmentation of retinal blood vessels is the first and a critical step for automated vessel analysis. After blood vessel segmentation, more advanced analysis can be performed, for example, investigating its diagnostic and prognostic values for eye diseases such as arteriosclerosis and hypertension. Besides, in order to obtain more accurate results in medical image segmentation tasks, we plan to extend the WA-Net structure to three dimensions (3D) because the three-dimensional images are becoming broadly used in healthcare settings. This would be a fruitful area for further work. More than this, in the future, we can also consider adding diagnostic text to images and building new models to automatically diagnose diseases.

## 6. Conclusion

The proposed WA-Net showed excellent performance in capturing detailed blood vessels and superior performance on retinal vessel segmentation tasks with proven generalization ability. It is a general, high-performance computing framework that does not require any handcrafted features. As a consequence, it has the practical clinical application value in the automatic diagnosis system and the potential to assist doctors in the diagnosis of fundus diseases.

## Figures and Tables

**Table 1:** Modules designed in WA-Net.

WDSR-A1	WDSR-A2	WDSR-A3	LASPP
3 × 3 × 64	3 × 3 × 128	3 × 3 × 256	3 × 3 × 128, ReLU, *d* = 1
ReLU	ReLU	ReLU	3 × 3 × 128, ReLU, *d* = 2
3 × 3 × 16	3 × 3 × 32	3 × 3 × 64	3 × 3 × 128, ReLU, *d* = 4
1 × 1 × 16	1 × 1 × 32	1 × 1 × 64	3 × 3 × 128, ReLU, *d* = 8

**Table 2:** Comparison of the performance among different methods on the DRIVE dataset.

Type	Method	Year	*F*1	Sens	Spec	Acc	AUC
Unsupervised	Roychowdhury et al. [37]	2015	——	0.7395	0.9782	0.9494	0.9672
	Azzopardi et al. [38]	2015	——	0.7655	0.9704	0.9442	0.9614
	Zhang et al. [39]	2016	——	0.7743	0.9725	0.9476	0.9636

Supervised	Li et al. [40]	2015	——	0.7569	0.9816	0.9527	0.9738
	Liskowski and Krawiec[17]	2016	——	0.7763	0.9768	0.9495	0.9720
	Chen [41]	2017	——	0.7426	0.9735	0.9453	0.9516
	Yan et al. [42]	2018	——	0.7631	0.9820	0.9538	0.9750
	R2U-Net [43]	2018	0.8171	0.7792	0.9813	0.9556	0.9784
	DCCMED-Net^a^ [24]	2019	——	0.7268	0.9912	0.9681	0.9819
	CE-Net [19]	2019	——	0.8309	——	0.9545	0.9779
	WA-Net (ours)	2020	**0.8222**	**0.7875**	**0.9813**	**0.9566**	**0.9794**

^a^The listed result is obtained by DCCMED-Net with batch size 32, which is the same as WA-Net.

**Table 3:** Comparison of the performance among different methods on the STARE dataset.

Type	Method	Year	*F*1	Sens	Spec	Acc	AUC
Unsupervised	Roychowdhury et al. [37]	2015	——	0.7317	0.9842	0.9560	0.9673
	Azzopardi et al. [38]	2015	——	0.7716	0.9701	0.9497	0.9563
	Zhang et al. [39]	2016	——	0.7791	0.9758	0.9554	0.9748

Supervised	Li et al. [40]	2015	——	0.7726	0.9844	0.9628	0.9879
	Liskowski and Krawiec[17]	2016	——	0.7867	0.9754	0.9566	0.9785
	Roychowdhury et al. [44]	2016	——	0.7720	0.9730	0.9510	0.9690
	Chen [41]	2017	——	0.7295	0.9696	0.9449	0.9557
	Yan et al. [45]	2018	——	0.7581	0.9846	0.9612	0.9801
	Dense U-Net [36]	2019	——	0.7914	0.9722	0.9538	0.9704
	WA-Net (ours)	2020	**0.8223**	**0.7740**	**0.9871**	**0.9645**	**0.9825**

**Table 4:** Comparison of the segmentation performance between datasets.

Dataset	Method	Year	Acc	AUC
STARE (trained on DRIVE)	Li et al. [40]	2015	0.9545	0.9671
	Yan et al. [42]	2018	0.9580	0.9678
	DUNet [18]	2018	0.9474	0.9571
	WA-Net	2020	**0.9564**	**0.9707**
	Li et al. [40]	2015	0.9486	0.9677
DRIVE (trained on STARE)	Yan et al. [42]	2018	0.9444	0.9568
	DUNet [18]	2018	0.9481	0.9718
	WA-Net	2020	**0.9478**	**0.9700**

## Data Availability

The two public open-source datasets used to support this study are available at http://www.isi.uu.nl/Research/Databases/DRIVE/ and http://cecas.clemson.edu/∼ahoover/stare/.
